# Multifunctional Profiling of *Moringa oleifera* Leaf Extracts for Topical Application: A Comparative Study of Different Collection Time

**DOI:** 10.3390/antiox12020411

**Published:** 2023-02-08

**Authors:** Anna Baldisserotto, Riccardo Barbari, Chiara Tupini, Raissa Buzzi, Elisa Durini, Ilaria Lampronti, Stefano Manfredini, Erika Baldini, Silvia Vertuani

**Affiliations:** 1 Department of Life Sciences and Biotechnology, Section of Medicines and Health Products, University of Ferrara, Via Fossato di Mortara 17–19, I-44121 Ferrara, Italy; 2 Department of Life Sciences and Biotechnology, Section of Biochemistry and Molecular Biology, University of Ferrara, Via Fossato di Mortara 74, I-44121 Ferrara, Italy

**Keywords:** *Moringa oleifera* leaves, natural antioxidants, UV filter, butyl methoxydibenzoylmethane, photostability, extracts, sunscreens

## Abstract

This research investigated plant extracts as a source of potential new actives in the nutritional, cosmetic, and pharmaceutical fields. *Moringa oleifera,* which is extensively known for its nutritional properties, has been investigated in this work by preparation, characterization, and evaluation of the antioxidant (FRAP, DPPH, ORAC, and PCL test), antifungal, photoprotective, and cytotoxicity profile against human melanoma Colo38 cell line of two different extracts (hydroalcoholic and methanolic) and one infusion of dry leaves collected from Paraguay in four distinct harvest times (February, March, April, and May 2017). The outcomes of this study highlight *Moringa oleifera* as a potential ally to counteract skin aging and oxidative stress, as indicated by the favorable antioxidant profile of the extracts and infusions of Paraguay, which was, in all cases, superior to that provided by the same plant species when collected from Senegal. Moreover, some samples were more efficient in preventing the photodegradation of UVA filter butyl methoxydibenzoylmethane (Avobenzone) compared to commercial filters, thus suggesting an interesting future role as natural additives in sunscreens.

## 1. Introduction

The natural environment provides a large variety of compounds suitable for medicinal use. Humankind has always searched for remedies in the vegetal world with the aim of maintaining or attaining a wellness state, at first by developing medical practices linked to the traditional and religious culture of every society and population, and later, with the advent of modern medicine, by searching for phytocomplexes and active compounds, finding inspiration for the development of drugs for various kinds of diseases.

Products of natural origin will become increasingly important in the future of the cosmetic and pharmaceutical industries, in line with rising conscious and eco-friendly choices concerning, among other things, sunscreens, UV filters, and, more generally, photoprotection. In fact, there are many natural compounds endowed with photoprotection capabilities, for example through direct UV filtration, or synergism with other filters, or by counteracting side effects of commercial UV filter such as photodegradation or photosensitization [1].

Antioxidant compounds such as flavonoids and polyphenols play a crucial role in preventing and/or quenching UV radiation-related ROS generation and could be considered as valid options to improve sun protection and to slow down photoinduced skin ageing [2]. Some antioxidant compounds were also reported to have a booster activity towards commercial UV filters such as butyl methoxydibenzoylmethane (Avobenzone) [3].

*Moringa oleifera* is a medicinal plant used in various traditional medical practices and widely described in the scientific literature. It belongs to the genus of the Moringaceae family, which comprises 13 species found in sub-continental India, Kenya, and north and north-east Africa. The species *Moringa oleifera* is original to the sub-Himalayan region of northern India, Bangladesh, Pakistan, and Afghanistan, but is now widespread and cultured for health and food purposes in the tropical and subtropical areas. The favorable growing conditions for *Moringa oleifera* are 25–35 °C, at an altitude of 500 m under direct sunlight, and in slightly acid or alkaline soil (pH 5–9). However, it can tolerate different environmental conditions such as high temperatures up to 48 °C, low temperatures, altitude, and a broad variety of soil types. It thrives best under a tropical insular climate, it grows well in the humid tropics or hot dry lands, and can survive pauper soils and drought [4,5].

It is called the “tree of life” or “miraculous plant” in several traditional medicines, as each fraction, including leaves and roots, is edible and rich in substances with valuable health and nutritional properties [6].

Due to its beneficial effects, it constitutes a major part of countless traditional ethnomedical practices, and the main uses reported concern inflammatory states, fertility problems, and lifestyle-related diseases [7,8,9,10,11].

More recently, there has been a substantial increase in the number of scientific articles reporting various potential applications of various portions of *Moringa oleifera*, including hypocholesterolemia, hypoglycemia, antimicrobic, anti-proliferative, antispasmodic, anti-inflammatory, and antioxidant activities [12,13].

*Moringa oleifera* is a precious source of polyphenols, and its phenolic and flavonoid fractions have been extensively identified and characterized [14,15,16].

Those natural antioxidant compounds are directly linked with the antioxidant activity of Moringa extract. Following the growing interest in the usage of natural antioxidants in commercial food supplements, recently, it has been reported that extracts of *Moringa oleifera* leaves were able to act as natural preservatives for food storage, being more effective in terms of both antioxidant and antimicrobial capacity than synthetic preservatives such as BHT (butylated hydroxytoluene) [17,18].

Dry leaves are rich in phenolic acids and flavonoids, such as quercetin and its disaccharide derivative rutin, along with chlorogenic acid and ferulic acid [19].

Those compounds have also raised interest for their potential application as adjuvants for topical formulation, due to their side activities supporting the efficacy of sunscreen [20,21,22].

Overexposure to UV rays is the primary cause of sunburn, oxidative stress, and consequent degenerative processes such as photo-aging, inflammation, and skin-related diseases, including erythema, hyperpigmentation, or skin cancer [23,24].

In particular, UVB radiation, due to the shorter wavelength and higher energy, is mainly responsible for UVB-induced sunburn or erythema; it is also highly carcinogenic as it is responsible for direct interaction with biological macromolecules and for DNA/RNA photoinduced damage [25]. UVA radiation is less energetic, but it is characterized by higher skin penetration and is the primary cause of skin ageing through photodegradative processes involving collagen fibers and hyaluronic acid [26]. In addition to this, UV exposure is related to a reduction in the inner antioxidant system response.

For these reasons, the antioxidant and antiproliferative activities, while exploring the multifunctionality profile of a new drug or natural extract, occupy a prominent position among the supplementary biological activities of a UV filter molecule or raw material [1,25,26].

Substantial skin photoprotective effects (antioxidant, anti-inflammatory, and anticancerogenic capacity) are attributed to many polyphenolic natural compounds, due to their structural features such as hydroxyl and aromatic moieties that generally increase the UV absorption capacity [27].

Topical application of those bioactive ingredients showed a decrease in UVB-induced skin sunburn events, suggesting and enforcing a definitive protective role for the skin from UV-related negative side effects [28].

The raising of scientific literature regarding the photoprotective potential of natural compounds, however, has not led to the approval of any natural sunscreen. The combination of commercial UV filters (organic or inorganic) and vegetal extracts, instead, is stating itself as an affirmed formulation trend, due to interesting reports of increased UV absorption, booster activity, and/or prevention of UV-related damage [1,29].

Taking this into account, the rationale of this work was to characterize and biologically evaluate three types of *Moringa oleifera* extract for their potential in the cosmetic/medicinal field, focusing the characterization on natural compounds that could play a significant role in photoprotection. Moreover, sun-related skin damages are emerging as a complex pattern of mechanisms that would benefit from multifunctional molecules. Thus, to complete the characterization of a multifunctional profile of these extracts, in view of a possible topical application, the antiproliferative activity on the human melanoma cancer cell line Colo38 was investigated as well in order to possibly identify chemopreventive properties. This further investigation is supported by several examples of the antiproliferative effects of extracts of different fraction of *M. oleifera* reported in the literature [30,31,32], and from our previous studies on *M. oleifera* from Senegal, which outlined an interesting activity of the hydroalcoholic extract in the low micromolar range [6].

Among the biological activities, *Moringa oleifera* extracts have been recently investigated against another important skin problem associated with the proliferation of fungi species [33,34,35]. Both the antiproliferative and antifungal activities could support the potential multifunctional profile of herbal extracts and are undoubtedly desirable complementary features in the topical application field. Because the content of active ingredients in plants largely depends on the environmental conditions (i.e., site of cultivation, altitude, latitude, longitude, etc.), in this study, we decided to investigate plant samples from the Asunción area, Paraguay, focusing on the effect of seasonality (ranging from late summer to early winter period) on qualitative and quantitative composition and biological activity.

## 2. Materials and Methods

### 2.1. General

All reagents and solvents were commercially available and used without further purification. The spectrophotometer used for antioxidant analysis was a Beckman Coulter™, DU^®^ 530, Life Science UV/VIS spectrophotometer, Single Cell Module (Beckman Coulter s.r.l., Via Roma, 108-Palazzo F1, Centro Cassina Plaza 20,060-Cassina De’ Pecchi, Milano, Italy). The instrument used to conduct ORAC analysis was a Fluoroskan FL^®^ ascent (Thermo Fisher Scientific, Inc., Waltham, MA, USA) with fluorescent filters (excitation wavelength: 485 nm; emission filter: 538 nm), linked to Ascent Software ^®^ (version 2.6) for data control and processing. In the sample loading phase, 96-well plates with a black background were used. PCL analyses were performed with a Photochem^®^ apparatus (Analytik Jena, Leipzig, Germany) with a Double Bore ^®^ phosphorus lamp (output 351 nm, 3 mWatt/cm^2^). The spectrophotometric analyses were conducted with a UV–Vis spectrophotometer SHIMADZU UV-2600 240 V. Photostability studies were performed with Atlas Suntest CPS+ solar simulator, (URAI S.p.a., Assago, Milano, Italy). WW5 PMMA plates were purchased from Schonberg GmbH (Munich, Germany). Microorganisms used in this study were *Epidermophyton floccosum* var. floccosum (The Netherlands), CBS 358.93 strain; *Trichophyton tonsurans* (The Netherlands), CBS 483.76 strain; *Trichophyton mentagrophytes* (The Netherlands), CBS 160.66 strain; *Microsporum canis* (Iran), CBS 131110 strain; *Microsporum gypseum* (Iran), CBS 130948 strain; *Arthroderma cajetani* (The Netherlands), CBS 495.70 strain; and *Candida albicans* (ATCC 10231). All dermatophytes were maintained at 4 °C as agar slants on SDA. Sabouraud dextrose agar (SDA) was purchased from Sigma-Aldrich SRL, Milano, Italy. HPLC analysis was performed using an Agilent 1100 Series HPLC System equipped with a G1315A DAD and with a Hydro RP18 Sinergi 80A column (4.6 × 250 mm, 4 µm) from Phenomenex.

### 2.2. Plant Material and Extraction Method

*Moringa oleifera* leaves were collected in 2017 in February (lot number: FEB2017B1), March (lot number: MARZO2017B1), April (lot number: ABRIL2017B2), and May (lot number: MAYO2017B3) at Yhaca-Guazu (25°58′55″ S, 56°29′47″ W, Guairá Department, Paraguay). After collecting, the leaves were dried by MANITS AGROPY S.A. (Josè Berges 988 esq.Peru–Asunción, Paraguay). Dried samples were packaged and sent to EDYNEA Srl (Viale dell’Industria, 32, Trissino, Vicenza), who supplied them to our laboratory. Before use, dried leaves were ground with a mortar until a fine powder was obtained, then stored at room temperature.

The extraction processes were conducted by making some changes to the protocols previously defined by Vongsak et al. [36] and Nouman et al. [37].

Hydroalcoholic extract: 10 g of dried leaf powder was placed for 1 h under magnetic stirring with 200 mL of hydroalcoholic solution (ethanol:distilled water, 70:30) at room temperature. The residue was then filtered and concentrated in vacuo to provide the desired hydroalcoholic dry extract.

Methanolic extract: The methanol dry extract was obtained by mixing about 5 g of powder with 100 mL of methanol and subjecting the mixture to 2 sonication cycles each of 40 min at 80%, followed by 9 min of centrifugation at 3500 rpm. The supernatant of the two cycles was pooled and concentrated in vacuo to obtain the desired dry methanol extract.

Infusion: The infusion was obtained by adding 10 g of dried leaf powder to 150 mL of distilled water previously brought to boiling conditions, and then left in infusion for 30 min. The infused solution was then filtered and lyophilized to obtain the desired dried infusion.

### 2.3. In Vitro Antioxidant Activities

#### 2.3.1. DPPH Radical Scavenging

The different extracts of *M. oleifera* were tested to quantify the scavenging activity of DPPH radicals according to a procedure described by Wang et al. [38]. This in vitro assay is ideal for phenolic compounds and allows one to determine the antioxidant capacity in a short time. This test evaluates the ability of an antioxidant to donate hydrogen to reduce the free stable radical DPPH into 1,1-diphenyl-2-picrylhydrazyl by measuring the absorbance at 517 nm of the solution containing the tested product which decreases and changes color from deep purple to light yellow after reaction with the radical. The radical inhibition percentage is calculated using the following Equation (1):

DPPH radical-scavenging capacity (%) = [1 − (A1 − A2)/A0] × 100%(1)

where A0 was the absorbance of the control (without sample), A1 was the absorbance in the presence of the sample, and A2 was the absorbance without DPPH.

The IC_50_ values were then calculated, defined as the amount of antioxidant needed to decrease the initial DPPH concentration by 50%. A total of 0.750 mL of extract solution at different concentrations was added to a DPPH methanolic solution (1.5 mL), and the absorbance was measured by a UV–Vis spectrophotometer. The IC_50_ values were determined by regression analysis of the data obtained at different sample concentrations and expressed as µg/mL and s.

#### 2.3.2. FRAP (Ferric-Reducing Antioxidant Power)

The activity of the extracts was evaluated following the FRAP method by Benzie and Strain [39], which evaluates the capacity of reducing ferric ions (Fe^3+^) into ferrous ions (Fe^2+^) in the presence of TPTZ (2,4, 6-tripyridyl-s-triazine) in an acidic environment. The ferric-tripyridyl-triazine complex (Fe(III)-TPTZ) is reduced to the ferrous form (Fe(II)) in the presence of an antioxidant, and this reduction corresponds to an intense blue color with a maximum absorption peak at 593 nm. The FRAP reagent is a mixture of 0.1 M pH 3.6 acetate buffer, 10 mmol/L TPTZ in 40 mmol/HCl and 20 mmol/L ferric chloride in a 10/1/1 ratio and is prepared immediately before analysis. A total of 1.9 mL of FRAP reagent was added to 0.1 mL of diluted sample (or solvent when running blank). All samples were incubated in the dark at 37 °C for 10 min, and then the absorbance was measured at 593 nm with a UV–Vis spectrophotometer. A calibration curve was prepared using Trolox as a standard and the results were expressed as µmol of Trolox equivalent (TE) per gram of dry matter.

#### 2.3.3. ORAC (Oxygen Radical Absorbance Capacity)

The ORAC test was performed on the basis of the previously modified Hong procedure [40]. In this assay, the peroxyl radicals generated by 2,2’-azobis (2-amidinopropane) dihydrochloride (AAPH) attack the target represented by the sodium salt of fluorescein (85 nM). A Trolox calibration curve (standard control) was performed at different concentrations (40–240 µM solutions). The tested extracts were solubilized in methanol or methanol–water mixture and then diluted in phosphate buffer solution pH 7.4 (PBS). The reading plate was incubated for 30 min at 37 °C and subjected to a fluorescence reading. After 30 min of incubation, the AAPH was added automatically, the plate was shaken for 5–10 s, and the fluorescence was kinetically monitored for 14 h. The ORAC values were calculated as the difference of the areas under the fluorescein quenching curves between the blank and the sample and were expressed as equivalent in µmol Trolox (TE) per gram of dry sample.

#### 2.3.4. PCL (Photochemiluminescence)

The PCL test is able to measure the antioxidant capacity of a sample against superoxide anion radicals. It is based on the method of Popov and Lewin [41] and quantifies the antioxidant capacity of a sample against superoxide anion radicals. The radicals are generated by Luminol, a photosensitizing agent, following exposure to UV light (Double Bore^®^ phosphorous lamp, output 351 nm, 3 mWatt/cm^2^). The antioxidant capacity was measured using the ACL kit (Antioxidant Capacity of the Liposoluble substance) of the manufacturer. The kinetic light emission curve was monitored for 180 s and the final result was expressed as micromoles of Trolox (standard) per gram of dry sample. The areas under the curves were calculated using PCL soft control and analysis software.

The extracts of *M. oleifera* were solubilized in methanol and suitably diluted before the analysis, while the formulations were suitably treated to extract the component to be tested. The antioxidant test was carried out in triplicate for each sample.

### 2.4. Total Polyphenol Quantification

An adapted and optimized Folin–Ciocalteu method was used to evaluate the total polyphenol content in *M. oleifera* extracts [42]. Gallic acid at different concentrations (0–500 ppm) was used as standard to obtain a calibration curve. A mixture consisting of Folin–Ciocalteu reagent diluted in water and extracts or gallic acid was incubated for 5 min at room temperature. Then, 300 µL of a sodium carbonate solution was added, and the whole mixture was incubated in the dark at room temperature for another 90 min. Finally, the absorbance at 765 nm was measured using a UV–Vis spectrophotometer against a blank containing distilled water in place of the extracts and the standard. The results of at least one triplicate are expressed as microgram gallic acid equivalents (GAE) per milligram of sample tested (µg GAE/mg dry extract).

### 2.5. Characterization of Polyphenols by HPLC

HPLC analysis was performed using an Agilent 1100 Series HPLC System equipped as describe above. The separation was monitored at an absorbance value of 254 ± 8 nm. The elution conditions, using water (0.01 M H_3_PO_4_) as solvent A and acetonitrile (0.01 M H_3_PO_4_) as solvent B, were programmed to gradient, using the following ratios: 90:10 (A/B) to 80:20 (A/B) in 5 min, maintained for 5 min, 80:20 (A/B) to 20:80 (A/B) in 10 min, 20:80 (A/B) to 90:10 (A/B) in 2 min, with a flow rate of 1.2 mL/min at room temperature. The injection volume for all samples and standards was 5 µL. Standard solutions of nicotinic acid, ellagic acid, rutin, chlorogenic acid, ferulic acid, and quercetin were prepared in methanol and properly diluted with methanol to obtain final concentrations between 80 and 0.625 µg/mL. An accurately weighed aliquot of each extract was dissolved in methanol or water–methanol (50:50). Before injection, each solution was filtered through a 0.45 μm nylon membrane filter and analyzed in triplicate by HPLC. The quantitative HPLC analysis was calculated, for each compound, according to its peak area.

### 2.6. Anti-Fungal Activity

#### 2.6.1. Anti-Dermatophyte Activity

The in vitro antifungal activity against the six selected dermatophytes was evaluated using the previously described plaque growth inhibition method [43]. Each extract was dissolved in DMSO. A suitable solution adjusted to 0.1% was added to the sterile culture medium (SDA) at 45 °C. The final concentration in the medium was 100 μg mL^−1^. The growth inhibition rates were calculated by comparing the mean value of the mycelium diameters in the control plates with that of the untreated control plates following Equation (2):

I = [(C − T)/C] 100%(2)

where I is the growth inhibition rate (%), C is the extended diameter of the control circle mycelium (mm), and T is the extended diameter of the test circle (mm). The percentage of growth inhibition was determined as the average of three different experiments.

#### 2.6.2. Anti-Candida Albicans Activity

*Candida albicans* suspension was prepared in an aliquot of 5 mL of sterilized water. Stock solutions were prepared by dissolving the extracts and fluconazole in DMSO at a concentration of 12.80 mg/mL. Minimal inhibitory concentration (MIC) of the extracts was performed by the broth microdilution method according to the Clinical and Laboratory Standards Institute/National Committee for Clinical Laboratory Standards (CLSI/NCCLS) Approved Standard M27-A3, 2008 (NCCLS) as previously described [44].

### 2.7. Formulations

A basic topical O/W formulation was prepared. The components of the hydrophilic phase were weighed and added in succession in a beaker as follows. Water and glycerin were brought to 60 °C. Xanthan gum and phenoxyethanol and ethylhexylglycerin were then added via a turbo emulsifier. The components of the oil phase were weighed separately and brought to a temperature of 70 °C, until solubilization of the ingredients. After that, the oil phase was slowly added to the aqueous phase under constant stirring with an ULTRA-TURRAX turbo emulsifier. Finally, *Moringa oleifera* samples (at 5% or 10%, depending on the combination) were added under stirring. In the case of the formulations containing Avobenzone, this was incorporated in the oil phase when the temperature was equal to 40 °C. The final pH of the formulations was brought to 6 with 10% NaOH.

INCI: Aqua, glycerin, xanthan gum, phenoxyethanol (and) ethylhexylglycerin, potassium olivoyl hydrolysed wheat protein, cetearyl alcohol, cocoglycerydes, coco-caprylate.

### 2.8. Evaluation of UV-Filtering Parameters

A precisely weighed amount of 0.0320 ± 0.0005 g of each formulation was spread in circular motions on 25 cm^2^ PMMA plates. The plates were weighed before and after the spread. Three plates were prepared for each product and five measurements were made of each plate. Before taking the measurement, the plates were located away from light, at room temperature, for 15–30 min. Finally, UV transmittance measurements from 290 to 400 nm were performed using a UV–Vis spectrophotometer. The blank was prepared using a plate coated with 15 μL of glycerin due to its non-fluorescent and UV transparency. The in vitro SPF, UVA-PF, and critical wavelength parameters were performed using the SPF Calculator Software (SPF Calculator Software (version 2.1), Shimadzu, Milan, Italy) as previously described [6].

### 2.9. Photostability

Each selected extract, alone or in combination with commercial UV filters, was incorporated in an oil-in-water (O/W) emulsion and spread on a PMMA plate. PMMA plate was irradiated with a solar simulator applying different UVA dose equivalents to an effective erythema radiant exposure of 150 kJ/m^2^. Before and after the exposure, the spectral transmittance of thin film of sunscreen was recorded from 290 to 400 nm. According to Equations (3) and (4), the residual percentages of SPF in vitro (% SPFeff.) and UVA-PF (% UVA-PFeff.) were calculated, respectively.

%SPFeff. = in vitroSPFafter/in vitroSPFbefore × 100(3)

%UVA PFeff = UVA PFafter/UVA PFbefore × 100(4)

### 2.10. Cell Proliferation Assays

The potential antiproliferative effects of the most interesting samples (hydroalcoholic, methanolic extracts, and infusion derived from *Moringa oleifera* leaves) were studied on two different human cell lines: the melanoma Colo 38 cells and the non-tumoral keratinocyte HaCat cells [6,45,46].

The melanoma Colo 38 cells were cultured in RPMI 1640 medium (Lonza, Verviers, Belgium), with 10% fetal bovine serum (FBS) (Biowest, Nuaillé, France) and 100 U/mL penicillin/streptomycin (Lonza, Verviers, Belgium); the pH of the medium was 7.2 and all the cell cultures were incubated at 37 °C in a 5% CO_2_ atmosphere. A total of 40,000 cells/mL were seeded in 24-well plates in RPMI complete medium. Twenty-four hours after seeding, compounds were added in serial dilutions to obtain different concentrations (0.5, 5, 50, 250, and 500 μg/mL) and incubated for another two days. After 24 and 48 h, the cell suspensions were mixed and counted with a Z2 Coulter Counter (Coulter Electronics, Hialeah, FL, USA).

The keratinocyte HaCat cells were maintained in DMEM (4.5 g/L glucose) (Euroclone, Milan, Italy) with 10% FBS (Biowest, Nuaillé, France), 100 U/mL penicillin/streptomycin (Lonza, Verviers, Belgium), and 2 mM glutamine (Gibco Thermo Fisher, Waltham, MA, USA); the medium pH was 7.2 and all the cultures were incubated at 37 °C in a 5% CO_2_ atmosphere. A total of 25,000 cells/mL were seeded in 24-well plates in RPMI complete medium. Eight hours after seeding, the compounds were added in serial dilutions to obtain different concentrations (0.5, 5, 50, 250, and 500 μg/mL), and incubated for another 24 h. Then, cells were detached, resuspended in fresh complete medium, and counted with a Z2 Coulter Counter (Coulter Electronics, Hialeah, FL, USA).

### 2.11. Statistical Analysis

Relative standard deviations and statistical significance (Student’s t-test; *p* ≤ 0.05) were given where appropriate for all data collected. One-way ANOVA and Least Significant Difference (LSD) post hoc Tukey’s honest significant difference test were used for comparing the bioactive effects of different samples. Statistical software STATISTICA 6.0 (StatSoft Italia s.r.l., Padova, Italy) was used for all computations.

## 3. Results and Discussion

### 3.1. Antioxidant Activity

The present work is in continuity with our research project concerning the investigation of the biological activities of *Moringa oleifera* leaf samples collected from different locations [6]. Given the growing interest in natural ingredients in cosmetic products, capable of quenching free radicals responsible for oxidative stress, we decided to deepen our knowledge of *Moringa oleifera*, focusing on a different harvest location, Paraguay. Specifically, extracts obtained from leaves collected in four different periods of the year (February, March, April, and May of 2017) were compared in order to evaluate the variation in the activity profile, both in qualitative and quantitative terms. From each sample, two extracts (hydroalcoholic and methanolic) and an infusion were prepared for a total of 12 lyophilized samples, which were then investigated for their potential antioxidant, antifungal, UV-filtering, and photostabilizing activity. Different methods are available to measure the antioxidant capacity of natural extracts, whose components can be more or less specific towards the distinct radical species. In this work, we decided to evaluate the antioxidant activity of the extracts and infusions by means of DPPH, FRAP, ORAC, and PCL tests to obtain a broad in vitro activity profile (Table 1).

The DPPH values indicated a greater antioxidant activity in the extracts harvested in February and March, and in particular, the hydroalcoholic extract. On the other hand, the three extracts harvested in May showed the lowest inhibition values but still with an adequate antioxidant capacity. Collectively, both the extracts and the infusion of *Moringa oleifera* from Paraguay showed a better antioxidant profile in comparison with our previous data obtained from leaf extract of *Moringa oleifera* from Senegal [6], but still quite lower than that of some other medicinal plants from India, Brazil, or Bangladesh [47,48,49].

The results obtained from the FRAP test are in line with those of the DPPH test. Again, the highest values were returned by the three extracts of February together with the hydroalcoholic extract of March. The lowest value obtained came from the infusion of March (384.56 ± 9.20 µmol TE/g), although it is superior when compared to the infusion of the leaves of *Moringa oleifera* native to Senegal (369.24 ± 27.52 µmol TE/g) [6].

The ORAC values showed a suitable activity profile of all the extracts with similar values. The hydroalcoholic extract of February, even in this case, had the best profile, with over twice the antioxidant activity of the extract of dried leaves of *M. oleifera* from Senegal (2942.8 ± 27.28 µmol TE/g) [6].

PCL data confirmed February extracts as the best antioxidant candidates, with antioxidant capacity values of 689.24 and 640.64 µmol TE/g for hydroalcoholic and methanolic extract, respectively. The results obtained from the extracts of the months of March, April, and May presented a strong profile as well. The hydroalcoholic extract of March, especially, showed an activity comparable, albeit slightly higher, to that previously reported for the infusion (512.1 ± 10.30 µmol TE/g) and hydroalcoholic extract (506.8 ± 3.19 µmol TE/g) of leaves of *M. oleifera* from Senegal. The lowest value relative to the antioxidant profile of *M. oleifera* of Paraguay was shown by the hydroalcoholic extract of the month of May (397.5 ± 14.14 µmol TE/g), which has still a better profile when compared with the lowest value obtained from the same plant from Senegal (methanolic extract, 367.1 ± 6.96 µmol TE/g).

The quantitative determination of the total content of polyphenols in the 12 extracts provided the data presented in Table 2.

The total polyphenol content of the extracts of *M. oleifera* leaves was found to be considerable—indeed, all the extracts showed a clearly better profile with respect to our previously reported data [6].

The hydroalcoholic extracts of February and March, followed by the methanolic extract of February, displayed the highest content of polyphenols, equal to 82.51, 75.16, and 73.64 µmol GAE/mg, respectively. Those results are in agreement with the antioxidant tests we carried out: the greater the total polyphenolic fraction detected, the greater the antioxidant capacity. The above results of the Folin–Ciocalteu test highlighted the presence of a polyphenolic component in the extracts and infusions of leaves of the four months of harvesting of *M. oleifera* from Paraguay.

### 3.2. Quantitative Estimation of Polyphenols

Characterization of the aforementioned extracts and infusion was performed through HPLC analysis, in order to identify and quantify compounds of interest. Our attention focused on phenols known for playing an active role in the field of photoprotection [20,21,22]: chlorogenic acid, ellagic acid, ferulic acid, rutin, and quercetin.

The quantitative estimation of the selected actives, according to peak area, is given in Table 3.

The results summarized in Table 3 show that ferulic and chlorogenic acid were the most present active ingredients in the vegetable extracts of *Moringa oleifera*. The hydroalcoholic extracts of February, March, and April were rich more than ferulic acid, while the infusions showed higher contents of chlorogenic acid, which indeed was the active with the highest percentage in the infusion of May. It should be noted that quercetin is present only in the hydroalcoholic and methanolic extracts of the month of May. This aspect highlights the different interactions between the phytohormones of the plant, light, water availability, and temperatures that may occur at various moments of the year. While no significant quantitative variation was noted among the four harvest times of *M. oleifera* from Paraguay, we found some interesting differences with the quantitative composition of *M. oleifera* leaf extracts from Senegal [6], where rutin was the predominant phenol detected (up to 1.58 ± 0.057% in the hydroalcoholic extract vs. 0.55 ± 0.013% of February hydroalcoholic extract from Paraguay) and quercetin content was higher (0.26 ± 0.001% vs. 0.02 ± 0.002% of the hydroalcoholic extract of May), while both ferulic acid and chlorogenic acid percentages were significantly lower (1.04 ± 0.059% and 0.3 ± 0.013%, respectively) [6].

### 3.3. Antifungal Activity

An antifungal is essential to fight skin infections, and the excessive production of radicals is the body’s self-defense mechanism. In this regard, many secondary plant metabolites have been used as actives against fungal infections caused by dermatophytes [50]. It was therefore decided to test the twelve extracts at a 100 µg/mL concentration against six dermatophytes (*Microsporum canis*, *Microsporum gypseum*, *Trichophyton tonsurans*, *Epidermophyton floccosum*, *Trichophyton mentagrophytes*, and *Arthroderma cajetani*) with the diffusion method in sabouraud dextrose agar (SDA). The results (data not shown) indicated *E. floccosum* as the mushroom that is most sensitive to the inhibitory activity of *Moringa oleifera* extracts, with discrete results obtained by the extracts of the months of February (inhibition values between 21.43% and 23.81%) and of March, except for the infusion, with values of 31.65% and 21.52% for the hydroalcoholic and methanolic extract, respectively. All the other data referred to a weak activity; for some dermatophytes, the treatment with the vegetal extract seems to have slightly stimulated, rather than inhibited, the fungus growth.

Furthermore, a possible inhibitory effect against *Candida Albicans* was evaluated, and MIC (minimum inhibitory concentration) was calculated in order to establish the potency of the samples. However, neither of them showed any activity against *Candida Albicans* in the range of 0.0244–12.48 mg/mL after 24 and 48 h (data not shown).

### 3.4. Antioxidant Activity of O/W Formulation (PCL Test)

After we established the antioxidant capacity of the raw extracts, being topical application the final aim of those materials, we carried out the PCL test on twelve O/W formulations containing 5% of each extract or infusion from the four harvest times considered in this work, in order to establish if the strong antioxidant parameters obtained from the extract were maintained once incorporated into a cosmetic product. The results are reported in Table 4 and antioxidant activity is expressed as µmol TE/g.

As reported, the antioxidant capacity of the extracts is retained when they are formulated in a cosmetic vehicle, with values that are in line with those reported for the raw extracts, taking final concentration into account. No substantial variation, as observed for the raw extracts, was detected among the four harvest periods, with the hydroalcoholic extract from February leading the ranking in terms of antioxidant performance.

### 3.5. In Vitro UV-Filtering Parameters Determination

The in vitro SPF determination was performed in order to evaluate the filtering profile of the extracts when included in suitable cosmetic formulations compatible with topical application. An O/W green emulsion was chosen as vehicle, and the hydroalcoholic and methanolic extracts and the infusion were incorporated at 5%. All tested samples returned SPF values among 2 (data not shown), which could be considered valuable in the context of vegetal extracts: this value means that 50% of UVB radiation is filtered by the finished product, and phytocomplexes are usually heterogeneous and contribute to sun ray protection through other complementary mechanisms (i.e., antioxidant activity, photostabilization, etc.).

### 3.6. Photostabilization Activity

Recently, some ingredients of sunscreens have emerged as potential boosters, being able to improve the total SPF value of some filters, also acting as photostabilizers, contrasting the degradation of unstable UV filters through quenching of the reactive radical species generated after UV irradiation [1].

In this context, we decide to explore the potential photostabilizing effect of the best *Moringa oleifera* extracts emerging for the above-mentioned studies (i.e., the two extracts and the infusion from February) towards the commercial UVA filter butyl methoxydibenzoylmethane (Avobenzone). This organic filter, although very active and inexpensive, is known to be photo-unstable due to keto-enolic tautomerism induced by UV irradiation, and must be used in combination with other UV filters that act as stabilizers, such as Octocrylene. These filters have both encountered several concerns in their application, being banned in the so-called “tropical paradise” of Haway and Palau for their negative impact on the marine environment and also for their skin irritation problems due to photosensitization. In the perspective of a “green” alternative, natural antioxidants may be promising options to improve Avobenzone photostability [3]. The two extracts and the infusion were incorporated at a 5% concentration in a suitable O/W formulation containing Avobenzone at a 5% concentration, while the same formulation with 5% Avobenzone and 5% Octocrylene as a stabilizer was used as a control. In addition, as ferulic acid is the active ingredient most present in February extracts, and known to be endowed with photoprotective action [22], we decided to further investigate its role as single potential photostabilizer in comparison to the whole extract. We therefore formulated Avobenzone at 5% with the exact percentage of ferulic acid present in the 5% concentration of the two extracts and the infusion from February, according to the above-mentioned HPLC characterization (0.19% for the hydroalcoholic extract and 0.09% for both methanolic extract and infusion).

To evaluate the efficiency of the extracts on the stability of Avobenzone, the formulations were subjected to SPF analysis before and after UV irradiation. According to regulatory criteria, if a filter shows a residual percentage of SPF in vitro and UVA-PF greater than or equal to 80, after irradiation, then it can be considered photostable [51]. The results are reported in Table 5 as %UVA-PFeff and %SPFeff after irradiation, given by the ratio of SPF/UVAPF value after irradiation and SPF/UVAPF value post-irradiation multiplied by 100, and %UVAPFdeg and %SPFdeg representing the percentage loss in terms of UVAPF and SPF value.

As expected, Avobenzone showed a marked photodegradation after UV exposure, with UVAPF and SPF values decreased by 22.33% and 16.23% respectively. Those data confirm its photo-unstable behavior in terms of UVA protection. The combination with Octocrylene (5%) furnished better post-irradiation filtering values, with 9.37% and 9.13% degradation of UVAPF and SPF values, respectively.

The two extracts and the infusion alone showed nearly quantitative photostability, with the slight exception of the methanolic extract, which showed degradation values below 5%.

The combination of the two extracts and the infusion with Avobenzone brought us some outstanding results: in all cases, the vegetal extract was able not only to completely avoid photodegradation, but in some way to improve the filtering parameters after irradiation (except for the methanolic extract with respect to the UVAPF value, which is near 100%). Even if it is not possible to define this behavior as a “booster” effect, those data collectively outline the potentialities of multifunctional herbal extracts as synergic agents with UV filters, and their ability to potentiate the photoprotection in terms of both SPF and UVAPF.

Finally, the combination of ferulic acid at the given concentrations and Avobenzone result in a slight decrease in the filtering parameters (2.92–5.44% for UVAPF and 4.01–4.27% for SPF value), corroborating the hypothesis that the stabilizing effect is due to a synergic effect of all the distinct components of the extract. Nevertheless, we found that ferulic acid was still able to inhibit photodegradation even at very low concentrations, if compared to the commercial filter Octocrylene—this confirms its ability to act as “enhancer” of anti-UVB and anti-UVA protection [52].

### 3.7. Anti-Proliferative Effects of Extracts on Human Melanoma Colo38 and Keratinocytes HaCat Cell Lines

With the aim to exclude possible side effects, *Moringa oleifera* leaf extracts and the infusion from February, which showed strong antioxidant activity and photostabilization capacity, were tested on the human melanoma Colo38 cell line and immortalized human keratinocytes HaCat cell line. Cells were seeded and cultured in the presence of increasing concentrations of *M. oleifera* extracts (0.05, 0.5, 5, 50, and 500 µg/mL). Then, after 24–48 h of treatment, cells were harvested and analyzed with a Beckman Coulter Counter. IC_50_ was calculated when cells were in the growth logarithmic phase, and indicates the drug concentration required for 50% cell growth inhibition. These preliminary results, summarized in Table 6, confirm the absence of evident antiproliferative effects on both cell lines (IC_50_ > 230.32 ± 18.90 µg/mL), also using the higher concentration of all the tested extracts on the Colo38 cell line and with the infusion treatment (IC_50_ > 500 µg/mL) on the HaCat cells.

## 4. Conclusions

Following the most recent trends in sunscreen formulation and UV protection and the increasing attention given to natural products able to play a part in skin photo-aging and oxidative stress [1,53], in this work, we evaluated the biological activities expendable in the pharmaceutic/cosmetic sphere of dry leaf extracts of *Moringa oleifera* from Paraguay, comparing the activity profile of four different harvest times (February, March, April, and May 2017) through three different extraction techniques (hydroalcoholic extract, methanolic extract, and infusion) for each month, with a total of 12 samples tested. Overall, all the tested extracts were superior in terms of antioxidant capacity and total polyphenol content when compared to the same plant from Senegal [6], with the two extracts from February and the hydroalcoholic extract from March leading the group. The antioxidant activity was also maintained when the extracts were incorporated in a suitable O/W emulsion for topical application at a 5% concentration. HPLC characterization returned chlorogenic acid and ferulic acid as the most present actives in all four months, while quercetin, which was detected in *M. oleifera* from Senegal, was detected in trace amounts only in the extracts of May, an aspect that highlights the variability in the production of secondary metabolites by the plant depending on several aspects that contribute to its growth (light, water availability, temperature, seasonality). The 12 extracts were tested for possible inhibitory growth activity towards six different dermatophytes and *Candida albicans*, but no inhibitory activity worth mentioning was obtained. Concerning the potential photoprotective activity and photostabilizing capacity of the extracts, values obtained from the SPF analysis of all the extracts tested were among 2, considered to be of interest due to other possible photoprotective mechanisms exerted by the vegetal extract. As proof of this, the combination of the extracts and the infusion from February and the organic UVA filter Avobenzone proved to increase the photostability of Avobenzone itself more efficiently than the commercial filter Octocrylene, returning UVAPF and SPF values after irradiation even higher than before irradiation. This is not to be considered a booster effect, but it points out the potential of extracts of *Moringa oleifera* leaves as solar formulation additives, both for their antioxidant and photostabilizing activity.

To summarize, *Moringa oleifera* leaf extracts from Paraguay can be considered as potential natural alternatives for sunscreen formulations, as proved by a set of biological activities, antioxidants and photostabilizers above all, that can synergistically improve skin photoprotection and reduce skin-related damages derived from excessive UV exposure. Considering the preliminary data on the antiproliferative effects on the HaCat cell line showed by the two extracts, the infusion from February emerges as the best candidate. Further studies will be conducted to establish the photostabilizing ability of these extracts even at lower concentrations than those considered in this work.

## Figures and Tables

**Table 1:** Antioxidant activity of *Moringa oleifera* leaf extracts collected in February, March, April, and May 2017. Each value is the mean of three different experiments (mean ± SE).

	Extract	DPPH (IC_50_) µg/mL	FRAP µmol TE/g	ORAC µmol TE/g	PCL µmol TE/g
	Hydroalcoholic	130.33 ± 3.84	900.07 ± 34.85	5959.06 ± 44.53	689.24 ± 7.30
February	Methanolic	138.61 ± 7.75	691.09 ± 48.48	4702.94 ± 34.18	640.64 ± 14.75
	Water	159.57 ± 10.93	557.24 ± 2.08	3830.72 ± 28.39	481.62 ± 5.20
	Hydroalcoholic	124.51 ± 1.27	739.29 ± 21.57	5346.63 ± 28.82	525.51 ± 0.00
March	Methanolic	180.01 ± 3.11	489.37 ± 7.80	4215.86 ± 9.23	416.67 ± 13.87
	Water	220.17 ± 7.43	384.56 ± 9.20	3343.76± 10.23	445.15 ± 12.63
	Hydroalcoholic	175.76 ± 11.31	551.39 ± 3.35	4556.42 ± 24.59	452.50 ± 14.14
April	Methanolic	218.54 ± 11.87	592.14 ± 33.26	4743.70 ± 15.63	472.36 ± 1.70
	Water	168.97 ± 3.10	561.75 ± 12.50	3813.07 ± 37.01	480.39 ± 17.33
	Hydroalcoholic	208.81 ± 6.29	516.37 ± 35.36	4956.35 ± 38.10	397.50 ± 14.14
May	Methanolic	203.37 ± 17.69	585.29 ± 29.42	4674.65 ± 44.47	445.31 ± 3.68
	Water	225.25 ± 1.71	457.11 ± 18.40	4131.49 ± 55.23	436.20 ± 9.21

**Table 2:** Total phenol content of *Moringa oleifera* leaf extracts. Each value was obtained from three experiments (mean ± SE).

	Total Phenol Content µg GAE/mg			
	February	March	April	May
Hydroalcoholic	82.51 ± 6.53	75.16 ± 6.04	67.62 ± 6.37	52.43 ± 2.37
Methanolic	73.64 ± 2.60	55.25 ± 6.35	65.95 ± 1.13	68.99 ± 7.04
Water	69.65 ± 10.93	59.01 ± 4.53	67.56 ± 5.27	61.24 ± 5.50

**Table 3:** Percentages of chlorogenic acid, ellagic acid, ferulic acid, rutin, and quercetin in the *Moringa oleifera* leaf extracts collected in February, March, April, and May 2017. Mean values obtained from at least three independent analyses (mean ± SE).

		Percentage of Detected Compounds (*w*/*w*)				
	Extract	Chlorogenic Acid	Ellagic Acid	Ferulic Acid	Rutin	Quercetin
	Hydroalcoholic	0.93 ± 0.084	0.82 ± 0.044	3.68 ± 0.016	0.55 ± 0.013	- ^a^
February	Methanolic	0.67 ± 0.012	0.85 ± 0.003	1.79 ± 0.111	0.58 ± 0.017	- ^a^
	Water	1.19 ± 0.119	0.35 ± 0.026	1.79 ± 0.018	0.30 ± 0.010	- ^a^
	Hydroalcoholic	0.92 ± 0.119	0.47 ± 0.033	3.04 ± 0.172	0.47 ± 0.029	- ^a^
March	Methanolic	0.67 ± 0.003	0.49 ± 0.019	1.53 ± 0.037	0.33 ± 0.015	- ^a^
	Water	0.96 ± 0.075	0.18 ± 0.006	1.38 ± 0.040	0.22 ± 0.005	- ^a^
	Hydroalcoholic	0.64 ± 0.045	0.44 ± 0.025	3.37 ± 0.200	0.29 ± 0.018	- ^a^
April	Methanolic	0.60 ± 0.050	0.50 ± 0.006	1.59 ± 0.166	0.28 ± 0.036	- ^a^
	Water	1.11 ± 0.099	0.21 ± 0.015	1.95 ± 0.027	0.20 ± 0.015	- ^a^
	Hydroalcoholic	1.02 ± 0.059	0.55 ± 0.052	1.94 ± 0.089	0.36 ± 0.012	0.02 ± 0.002
May	Methanolic	0.95 ± 0.070	0.69 ± 0.049	1.13 ± 0.090	0.34 ± 0.031	0.02 ± 0.004
	Water	1.13 ± 0.012	0.24 ± 0.014	1.05 ± 0.035	0.21 ± 0.008	- ^a^

^a^ Not detected.

**Table 4:** PCL test values for the twelve O/W emulsion with 5% of each extract/infusion. Results are the mean value of at least three independent experiments.

Harvest Time	Extract	PCL (µmolTE/g) 5% O/W Emulsion
	Hydroalcoholic	47.25 ± 3.12
February	Methanolic	33.66 ± 0.54
	Water	29.61 ± 0.54
	Hydroalcoholic	35.03 ± 0.54
March	Methanolic	32.13 ± 1.41
	Water	28.26 ± 1.18
	Hydroalcoholic	38.28 ± 0.63
April	Methanolic	27.31 ± 0.41
	Water	33.87 ± 1.53
	Hydroalcoholic	34.72 ± 1.89
May	Methanolic	32.13 ± 1.33
	Water	29.19 ± 1.18

**Table 5:** %UVAPFeff, %SPFeff, %UVAPFdeg, and %SPFdeg values for O/W emulsion after irradiation.

Emulsion O/W	%UVAPFeff	%SPFeff	%UVAPFdeg	%SPFdeg
Avobenzone 5%	77.67	83.77	22.33	16.23
Avobenzone 5% + Octocrylene 5%	90.63	90.87	9.37	9.13
Hydroalcoholic extract (February) 5%	100	100	0	0
Hydroalcoholic extract (February) 5% + Avobenzone 5%	110.32	109.21	−10.32	−9.21
Methanolic extract (February) 5%	95.03	96.67	4.97	3.33
Methanolic extract (February) 5% + Avobenzone 5%	99.58	111.74	0.42	−11.74
Infusion (February) 5%	99.30	98.61	0.70	1.39
Infusion (February) 5% + Avobenzone 5%	109.98	106.91	−9.98	−6.91
Ferulic Acid 0.09 % + Avobenzone 5%	94.56	95.99	5.44	4.01
Ferulic Acid 0.19% + Avobenzone 5%	97.08	95.73	2.92	4.27

**Table 6:** Antiproliferative effects of *Moringa oleifera* leaf extracts on human melanoma Colo38 and keratinocyte HaCat cell lines.

*Moringa oleifera*	IC_50_ (Colo38)	IC_50_ (HaCat)
Hydroalcoholic extract	>500 µg/mL	367.01 ± 24.58
Methanolic extract	>500 µg/mL	230.32 ± 18.90
Infusion	>500 µg/mL	>500 µg/mL

Data (mean values ± SD) were obtained from three independent experiments.

## Data Availability

Data are contained within the article.
